# Antimicrobial Resistance Elements in Coastal Water of Llanquihue Lake, Chile

**DOI:** 10.3390/antibiotics13070679

**Published:** 2024-07-22

**Authors:** Javier Campanini-Salinas, Catherine Opitz-Ríos, John A. Sagredo-Mella, Danilo Contreras-Sanchez, Matías Giménez, Paula Páez, María Clara Tarifa, Nataly D. Rubio, Daniel A. Medina

**Affiliations:** 1 Laboratorio Institucional, Universidad San Sebastián, Puerto Montt 5501842, Chile; javier.campanini@uss.cl (J.C.-S.); copitzr1@correo.uss.cl (C.O.-R.); jsagredom@correo.uss.cl (J.A.S.-M.); nconchar@correo.uss.cl (N.D.R.); 2 Facultad de Medicina y Ciencia, Universidad San Sebastián, Puerto Montt 5501842, Chile; dcontreras@docente.uss.cl; 3 Laboratorio de Genómica Microbiana, Institut Pasteur Montevideo, Montevideo 11400, Uruguay; mgimenez@pasteur.edu.uy; 4 Centro de Investigaciones y Transferencia de Río Negro, Universidad Nacional de Río Negro, Villa Regina 8336, Argentina; ppaez@unrn.edu.ar (P.P.); mctarifa@unrn.edu.ar (M.C.T.); 5 Centro de Investigaciones y Transferencia de Río Negro, (CIT Río Negro, UNRN-CONICET), Villa Regina 8336, Argentina; 6 Escuela de Medicina Veterinaria, Facultad de Ciencias de la Naturaleza, Universidad San Sebastián, Puerto Montt 5501842, Chile

**Keywords:** antimicrobial resistance, metagenomics, DNA mobile elements

## Abstract

Antimicrobial resistance has been stated to be a global health problem. In Chile, the use of antibiotics should be declared by medical prescription, but it is unknown what happens to the drugs once the treatment ends. Among the possibilities for their disposal are the trash or the drain; regardless of which scenario arises, antibiotics could accumulate in the environment, stimulating the emergence of antimicrobial resistance mechanisms and their transfer between microorganisms. Unfortunately, sometimes wastewater ends up in bodies of water, due to the dragging of elements by rain, or by the presence of illegal water discharges. In this work, shotgun metagenomics was used to elucidate the functional and microbial composition of biohazard elements in the bay of Puerto Varas City, Chile. As expected, a high diversity of microorganisms was found, including bacterial elements described as human or animal pathogens. Also, a diverse repertory of antimicrobial resistant genes (ARGs) was detected, which confers mainly resistance to macrolides, beta-lactams, and tetracyclines, consistent with the families of antibiotics most used in Chile. Similar ARGs were identified in DNA mobile elements. In addition, we tested the antimicrobial susceptibility in 14 bacterial strains isolated from Llanquihue Lake. This is the first report of the presence of genomic elements that could constitute a health problem, considering the importance of the interconnection between environmental, animal, and human health, a concept known as One Health.

## 1. Introduction

The emergence and spread of antimicrobial resistance (AMR) is a major global health problem [1] and the World Health Organization (WHO) has classified it as a major threat to global public health [2]. AMR occurs when microbes, such as bacteria, fungi, viruses, and parasites, become resistant to the drugs used to treat them [3], hindering or even making it impossible to treat infections caused by antimicrobial resistant microorganisms [4]. The widespread implications of AMR extend beyond individual health outcomes to encompass broader public health and economic challenges [5]. AMR has economic consequences; its impacts include increased healthcare costs, lost productivity, and overall economic burden [6], reflected mainly in an increase in healthcare costs to treat persistent microbial infection due to extended treatment, and the clinical readmission of unhealed patients [7]. Antimicrobial resistance can lead to increased costs of treating resistant bacterial infections. However, the economic burden goes beyond healthcare costs and includes reduced income due to prolonged illness and premature death, affecting both individuals and society [8]. In low- and middle-income countries, where infectious diseases are most prevalent, the failure of first-line antibiotics has led to increased mortality and costs [9]. In addition, the economic impact of antimicrobial resistance affects not only humans but also animals, leading to economic losses in animal husbandry and further straining economies [10].

Many factors contribute to the development and spread of AMR, including the overuse and misuse of antibiotics in human and animal clinical practice, the indiscriminate use of antibiotics in animal production as growth promoters, poor sanitation and hygiene of health care systems, and the improper disposal of waste contaminated with antibiotics and resistant bacteria [11]. One of the leading causes for the dissemination of AMR is the presence of antimicrobial resistance genes (ARGs) in environmental reservoirs, such as lakes, rivers, and oceans [12]. Also, mobile genetic elements (MGEs) play a significant role in the transference and dispersion of ARGs among bacteria. They facilitate horizontal gene transfer (HGT) by several mechanisms. MGEs can carry ARGs and transfer them to other bacteria in genomic elements known as plasmids, contributing to the spread of ARGs [13]. Understanding the prevalence and distribution of ARGs and MGEs in aquatic ecosystems is crucial to develop strategies that mitigate their potential impact on public health. The resistome consists of all ARGs, including those circulating in both pathogenic and non-pathogenic bacteria [14], and aquatic environments have been already reported as reservoirs of these ARG elements [15,16].

Given the significant increase in global anthropogenic activities and the growing concern regarding AMR, it becomes imperative to investigate the presence and characteristics of ARGs and MGEs in the environment. By investigating the presence of ARG elements in coastal water recovered from Puerto Varas shore, this study aims to shed light on the prevalence, distribution, and potential implications of antimicrobial resistance in this specific aquatic environment. This research is essential for developing strategies to mitigate the spread of antimicrobial resistance and safeguard public health in the region.

Shotgun metagenomics is a powerful tool that allows us to sequence in depth all the DNA present in a sample, enabling the exploration of the genetic and functional diversity of microbial communities [17]. In this work, we used shotgun metagenomics to perform a quick description of the microbial composition of Llanquihue Lake at three points of Puerto Varas city shore, aiming to characterize the bacterial communities present in the beach and identify the existence and abundance of ARGs in the environmental DNA due to the important role of these biologic elements for human health. By assessing the ARGs and identifying the MGEs that may drive ARG transference, this research will help to understand the hazards associated with the transfer of AMR from environmental reservoirs to humans. This work constitutes an example of how metagenomics can be useful in the surveillance of microbiological risks in areas where cities are in close contact with the natural environment.

## 2. Results

### 2.1. Composition of Bacterial Communities That Inhabit Llanquihue Lake

**Figure 1:**
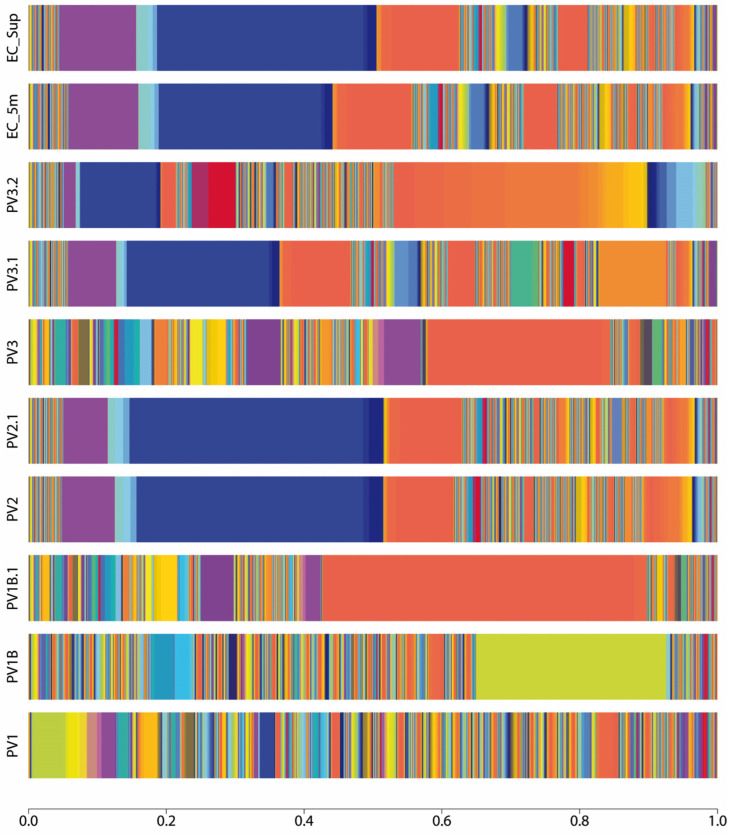
Taxonomy abundance at the species level represented as stacked bar plot of each sample site of Puerto Varas shore. Three points of the Llanquihue lake beach were sampled (PV1, PV2, PV3) and one point (EC) 200 m far from the coast was sampled, at surface (SUP) and 5 m submerged from the water column (5 m). The sampling was repeated one month later (samples namely with .1). Only PV3 sampling was repeated two months later (namely .2). The color pattern of each bar shows the microbial community structure, while the amplitude of each color represents the percentage of abundance of the assigned taxonomy. The blue color that dominates the taxonomical pattern in PV2, PV2.1, PV3.1, and EC samples belongs to “*Candidatus Nanopelagicus abundans*”, while the orange color in PV1B.1 and PV3 represents the taxa *Prevotella copri*. In PV1B, the olive color represents the abundance of the taxa, namely *Stenotrophomonas maltophilia*.

**Figure 2:**
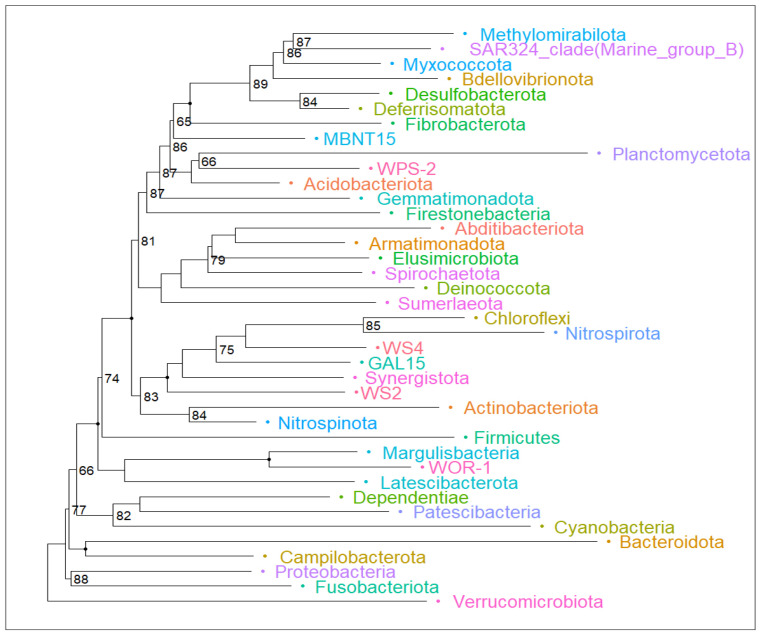
Phylogenetic tree that reveals the intricate web of relationships among microbial phyla. Major phyla such as Verrumicrobiota, Fusobacteria, Proteobacteria, Firmicutes, Actinobacteria, and Bacteroidetes emerge as prominent branches, highlighting their importance in processes such as fermentation and natural decomposition. The number denotes the bootstrap value of each node below a confidence value of 90 percent. Non-numbered branches have a bootstrap value above 90 percent.

Shotgun metagenomic sequencing and bioinformatic analysis indicate a heterogeneous taxonomic composition of bacterial-type microorganisms in Llanquihue Lake (Figure 1, Supplementary Figure S1, and Supplementary Table S1), indicating the taxonomic complexity associated with the microbial communities that inhabit the lake (Figure 2). Taxonomical assignation shows that the main phyla presented belong to Proteobacteria, Firmicutes, Bacteroidota, Actinobacteriota, and Verrucomicrobiota (Figure 2 and Supplementary Table S1). In the taxonomic data, the presence of bacterial genera *Brucella*, *Mycoplasma*, *Mycobacterium*, *Microcystis*, and *Flavobacterium* was identified, which harbor bacterial species of interest in veterinary clinical practice [18]. In addition, we found the presence of bacterial genera belonging to intestinal microbiota such as *Prevotella*, *Coprococcus*, *Bifidobacterium*, *Faecalibacterium*, and *Ruminococcus* [19], with *Prevotella copri* being one of the most abundant species in sample PV1.1. At the species level, a total of 3740 species (Supplementary Table S1) were identified, including environmental water-related species such as *Nanopelagicus abundans* [20], *Fonsibacter ubiquis* [21], and *Planktophila vernalis* [22]. Interestingly, several taxonomical species related to skin and intestinal infections in humans were detected, mainly belonging to the genus *Campylobacter*, *Clostridium*, *Escherichia*, *Mycobacterium*, *Salmonella*, *Shigella*, *Staphylococcus*, *Streptococcus*, and *Yersinia* (Supplementary Figure S1). Although many of these taxonomic findings are repeated among the analyzed sites, the abundance of each of these taxonomies varies, indicating that the microbial community structure differs depending on its location (Figure 1). In specific, some of the bacterial species identified can be related to the presence of birds (*Jeotgalibaca ciconiae*, *Ornithobacterium rhinotracheale*, *Pasteurella multocida* subsp. *multocida*, and *Riemerella anatipestifer*), while others can be related to the presence of wild fish, marine birds, and aquaculture activities performed around the lake (*Aeromonas salmonicida*, *Vibrio anguillarum*, *Flavobacterium columnare*, *Flavobacterium psychrophilum*, *Renibacterium salmoninarum*, *Aliivibrio fischeri*, *Piscirickettsia salmonis*, and *Tenacibaculum maritimum*). Also, we detected the presence of DNA sequences related to bacteria genus belonging to soil and vegetation (*Rhizobium*, *Streptomyces*, and *Mesorhizobium*). Overall, these observations reflect the complex composition of the microbial community present in Llanquihue Lake.

### 2.2. Identification of AMR Genes Present on DNA Recovered from Llanquihue Lake

**Figure 3:**
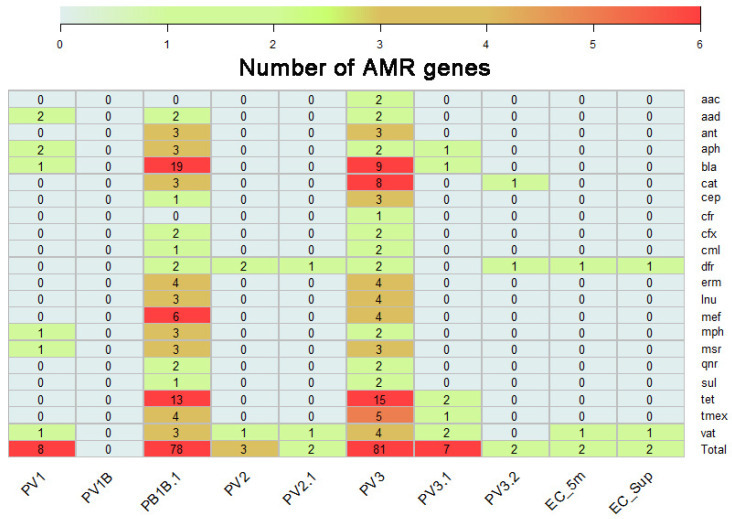
AMR gene families identified on environmental DNA recovered from Llanquihue Lake. The heatmap represents the genes identified and groups them according to their antimicrobial resistance family.

The metagenomic analysis revealed a diverse array of antimicrobial resistance genes across the environmental DNA recovered from water samples. To report this, we group the different genes identified according to the antimicrobial resistance family to which they belong (Figure 3). These included genes that confer resistance to antibiotics commonly used in human clinical treatments, such as β-lactams and chloramphenicol, as well as genes associated with resistance to antibiotics used in agriculture and veterinary medicine, such as macrolides, tetracyclines, and fluoroquinolones, such as the *tet* genes, which confer resistance to tetracyclines by encoding for efflux proteins, or by encoding ribosomal protection proteins or enzymes that chemically modify tetracycline [23]. Another remarkable family of genes with high prevalence was the *bla* family, which encodes resistance to beta-lactam antibiotics [24,25], as well as the *dfr* genes, which encode the trimethoprim-resistant dihydrofolate reductase, initially found in *Escherichia coli*, *Salmonella enterica*, and *Pasteurella multocida* [26,27,28], and *cat* genes, which encode for chloramphenicol acetyltransferase for the inactivation of chloramphenicol by addition of an acyl group [29]. Interestingly, we detected the presence of *mcr*-7.1 sequences in one sample, which confers resistance to colistin [30]. Notably, the abundance and composition of ARGs varied spatially and temporally, with differential presence in areas impacted by anthropogenic activities, such as rainwater drainage (PV1 area and its time replicates) and public beaches (PV2 and PV3 areas, and its time replicates). Table 1 summarizes the antimicrobial family gene, a pharmaceutical drug example, and includes an example mechanism that confers the described resistance.

### 2.3. Mobile Genetic Elements Carrying AMR Genes Are Related to Microbial Species of Health Interest

To further complete the characterization of ARGs present in the Llanquihue lake, metagenomic assemblies were screened to explore the presence of MGE. The results showed the presence of mobile elements belonging mainly to the MOBP1 group, classified based on their relaxase gene [38], which included elements identified in bacterial host belonging to genus *Clostridiales*, *Clostridioides*, *Aeromonas*, *Vibrio*, *Enterococcus*, *Escherichia*, *Bacteroides*, *Parabacteroides*, *Klebsiella*, and *Proteus* (Supplementary Table S2). However, a significant portion of the mobile elements were not classified according to a MOB group, such as pR997, pSX2_LC6, pRIVM_C010068_1, pAFAEC, and pMMCAT_PdisCL06T03, originally identified in hosts such as *Proteus mirabilis*, *Shewanella* sp., *Enterobacter hormaechei*, *Aliarcobacter faecis*, and *Parabacteroides distasonis*, respectively. Furthermore, we identified an overlap between the original host reported for MGE identified in our metagenomic data and the presence of harmful microbial species (Supplementary Figure S1 and Supplementary Table S2).

Because MGEs play a significant role in the evolution and adaptation of organisms by facilitating genetic diversity and horizontal gene transfer, we wondered which genes were being carried by the plasmid sequences identified. For this purpose, we explore MGE sequences (Supplementary File S1) to look for the specific presence of ARGs in its genomic code. As a general trend, we found ARGs conferring resistance to erythromycin, azithromycin, lincomycin, doxycycline, tetracycline, amoxicillin, ampicillin, mainly encoded by the genes *mph*(E), *msr*(D), *mef*(A), *erm*(B), *erm*(F), *lnu*(C), *tet*(M), *tet*(W), *tet*(C), *tet*(O), *bla*SHV-12, and *ant*(6), respectively. These ARGs were carried in the MGE sequences (Supplementary File S1) identified initially in bacteria belonging to species such as *Lactococcus garvieae*, *Enterococcus faecalis*, *Acinetobacter* sp., *Escherichia coli*, and *Shewanella* sp.

### 2.4. Antimicrobial Susceptibility Tests of Microbial Isolates Do Not Show the Presence of Antimicrobial Resistance Patterns

A total of 14 different isolates of enterobacteria were recovered and cultured from water samples. A total of three isolates belonging to *Citrobacter* spp., three of *Enterobacter* spp., six of *E. coli*, and one of *Rahnella aquatilis*, were identified by 16S rRNA PCR, Sanger sequencing, and BLAST. Six antibiotics were tested on all isolated bacteria, and the inhibition halos obtained ranged from 15 to 49 mm. No resistant bacterial populations according to CLSI classification were detected (Table 2).

## 3. Discussion

Currently, studies with a One Health perspective, which aim at environmental, human, and animal surveillance, are needed due to the threat associated with AMR phenomena. It is crucial to understand that this phenomenon is ubiquitous; therefore, research must be conducted on humans, animals, and the environment as a whole. Detection of a high abundance of ARGs in different environments corresponds to one of the first steps required to counteract this phenomenon. Nevertheless, as cultivable bacteria only represent a small fraction of the whole microbiota within a specific environment [40,41], ARG monitoring mostly depends on studies performed from total DNA extracts [42]. Studies related to ARGs in a variety of environmental areas have been supported by molecular biology-based methods and sequencing methods [43,44,45,46,47]. Here, we studied the presence of ARGs in Llanquihue Lake through metagenome sequencing. As metagenomics is a non-targeted method for detecting and quantifying taxonomic and functional genetic diversity in each environment, these strategies allow us to make inferences about the occurrence and proportions of a variety of groups within a complex microbial community [48]. In addition, metagenomics is one of the most attractive tools for exploring natural environments due to the large amount of information that can be obtained [49,50,51].

We could evidence the presence of *cfx*A6 and *cfx*A2 genes in Llanquihue Lake, related to the expression of class A beta lactamases, which have both cephalosporins and penicillins as substrates. In China, the presence of these genes has been detected in different water bodies [52]. The presence of these genes was also found in Poland, in a wastewater treatment plant [53]. In this study, we have found genes associated with the *bla* family: *bla*FAR-1, *bla*OXA-490, *bla*OXA-491, *bla*TEM-102, and *bla*TEM-104. These genes are also associated with the expression of beta-lactamases in different pathogenic bacteria. No reports were found in the literature about the presence of *blaFAR*-1, *blaOXA*-490, and *bla4*91 genes in water bodies. Furthermore, some reports indicate the presence of *blaT*EM-102 and *bla1*04 genes, which have previously been reported in different types of water bodies, and studies have mentioned the potential risk to human health posed by their presence in the environment [54,55,56].

Interestingly, we observed the presence of the *mcr*-*7* gene, whose family is related to conferring colistin resistance, a highly relevant drug in the treatment of infections complicated by multiresistant Gram-negative bacteria [57,58,59]. Some studies demonstrate the presence of these genes in water bodies around the world [60,61]. The presence of types of genes in a lake where recreational activities are undertaken constitutes a risk for the population. Abundant gene families found in Lake Llanquihue were the ones associated with resistance to tetracyclines such as *tet*(37), *tet*(A), *tet*(C), *tet*(O), *tet*(Q), and *tet*(W). Several studies have shown the presence of these resistance genes in aquatic environments [62,63,64,65]. For example, the *tet*(37) gene family has been reported at the environmental level in an anthropogenically stressed estuary on the northwest coast of Portugal [66]. In particular, the presence of these genes constitutes risks for productive activities associated with aquaculture. This is because one of the most widely used antibiotics in freshwater production cycles is oxytetracycline [67]. The potential expression of these genes in pathogenic bacteria affecting farmed fish could cause the ineffectiveness of these treatments. In summary, the presence of these genes in DNA isolated from water reservoirs highlights the widespread distribution of antimicrobial resistance determinants in the environment. Additionally, our analysis unveiled the presence of MGE, such as plasmids associated with ARGs, highlighting the dynamic nature of AMR in aquatic ecosystems and its potential dissemination.

One limitation of our study was to isolate and characterize bacterial strains with antimicrobial resistance phenomena. As detection of the presence of ARGs from data obtained by sequencing does not necessarily imply the expression of such genes in each microbial community, further empirical tests are required for describing the putative expression of resistant phenotypes [68]. To achieve this, we performed susceptibility assays in 20 microbial isolates obtained from Llanquihue Lake. Although the presence of multiresistant strains was not detected, as was expected concerning the metagenomic results presented here, these results might not be entirely representative of the occurrence of multiresistant strains in the environment. ARG detection through metagenome sequencing gives information about both culturable and unculturable bacteria; thus, classic microbiological techniques for culture and susceptibility assays from microbial isolates have limitations that should be considered for the detection and isolation of multiresistant strains.

Proper watershed management has important positive effects on the mitigation of human health risks associated with the presence of ARGs in the environment [69]. The implementation of effective public politics on water management, such as regulating and monitoring the discharges of domestic, industrial, and hospital wastewater into water bodies, can help to avoid ARG transference and pharmaceutical dispersion [70,71]. For example, reducing the use of agricultural antibiotics close to water bodies [72] or limiting the presence of aquaculture activities in freshwater [73] would contribute to reducing the load of pharmaceutical pollutants in water systems. Additionally, constant monitoring of water quality would contribute significantly to decision making to promote sustainable practices in the watershed [74]. Moreover, evidence-based decision making on water quality, supported by monitoring data, can be crucial for delivering safe drinking water, optimizing water quality, and managing water resources effectively [75,76]. By minimizing water pollution, selective pressure on harmful microorganisms that inhabit the aquatic environment would be reduced, diminishing the spread of antimicrobial resistance and virulence genomic elements [77,78]. This comprehensive approach would not only protect the health of local communities by safeguarding the purity of the water resource but would also contribute to the preservation of antibiotic effectiveness and sustainable public health management in the long term [79].

## 4. Materials and Methods

### 4.1. Sample Collection and Microbial Isolation

To obtain the microbial DNA, the water samples were collected from the coastal shore of Puerto Varas city, located close to Llanquihue Lake. Llanquihue Lake is the second largest lake in Chile, with a surface area of approximately 860 km^2^ and a maximum registered depth of 317 m [80]. Its main city, Puerto Varas, is on the lake’s eastern shore. The samples were taken from 3 sites of Puerto Varas shore, at a depth of 50 cm to the surface, at sites PV1, PV2, and PV3 (Supplementary Figure S2), on the shores of the city’s eastern beach, in front of the city center coast, and on the beach located at the west end of the city, respectively. The sampling was repeated one month later (samples namely with .1). Only PV3 sampling was repeated two months later regarding the first samples (namely .2). A total of 3 L of water for each sample site was collected using sterile 1 L glass bottles and preserved with an icepack until processed in the laboratory on the same day of sampling. A total of 3 L of water was filtered through mixed cellulose ester (MCE) membranes of 0.22 µm pore size (Merck-Millipore #GSWP04700, Burlington, MA, USA), using a glass filter system pumped with negative pressure. Filters were stored in RNA Later (Sigma-Aldrich #R0901, Saint Louis, MI, USA) until DNA extraction, as described below. In parallel, 1 mL of water was streaked on Brain Heart Infusion agar, Trypto-Casein Soy agar, Eosin Methylene blue agar, Mueller–Hinton agar, and MacConkey agar plates, and cultivated at 25 °C for 24 h. Isolated cell colonies grown in each media were passed 2 times to new agar plates of the same media to facilitate their purification, and then, Gram stain was used to check the purity of the isolated bacteria. Isolated microorganisms were observed under microscopy and stored in sterile glycerol 10% *v*/*v* at −80 °C.

### 4.2. DNA Purification and Metagenomic Sequencing

The stored MCE filters were used for DNA extraction employing AccuPrep Genomic DNA Extraction Kit (Bioneer #K-3032, Daejeon, Republic of Korea), following the manufacturer’s instructions. Briefly, filters were resuspended in 500 µL DNA Extraction buffer and stirred to release microbial cells. Enzymatic digestion with 20 µL of lysozyme (20 mg/mL) and 20 µL of proteinase K (20 mg/mL) was used to disrupt microbial cells. The suspension was incubated for 1 h at 37 °C and then for 1 h at 55 °C. After enzymatic digestion, we followed the steps provided by the manufacturer for bacterial DNA extraction. The quality of the obtained DNA was checked by 1% agarose gel electrophoresis, while DNA quantity was measured by absorbance and the ratios 260/280 nm were calculated to assess the purity of the DNA obtained. Before DNA sequencing, we tested the amplification capacity of DNA using 16S bacterial universal PCR. A total of 1 µg of DNA was sent to Novogene (Sacramento, CA, USA) genomic service for shotgun metagenomic sequencing. DNA was sequenced by paired-end (2 × 150 bp) reads using the Illumina NovaSeq 6000 (San Diego, CA, USA) platform with an output of 6 GB per sample.

### 4.3. Metagenomic Data Analysis and Identification of AMR Genes

Raw data obtained from the sequencing provider were initially inspected with FastQC (https://www.bioinformatics.babraham.ac.uk/projects/fastqc, accessed on 15 December 2023), and then reads were filtered and trimmed using Trimmomatic v0.40 [81] using the following parameters: LEADING:20, TRAILING:20, SLIDINGWINDOW:5:20, AVGQUAL:20, and MINLEN:90, followed by the application of Bowtie2 to screen out the contaminant DNA sequences from human and viruses [82]. The paired-end files were merged using the script provided in the Microbiome Helper v2.3 pipeline [83] and metagenomic data were processed to obtain metagenomics de novo assembly using MegaHit v1.2.9 [84], and the quality of the conting obtained was inspected using Quast v5.2 [85]. The taxonomic profiling was obtained at the species level using Kraken2 [86], keeping the taxonomic assignation with over 500 hits by sample, while antimicrobial resistance genes were inspected using ABRicate v1.0 [87], utilizing the Resfinder v4.5.0 [88] databases. Mobile genetic elements were retrieved using plaSquid v1.0.0 [89]. The fasta files obtained from plaSquid were used to look for the presence of AMR genes carried in the mobile elements using ABRicate, as described above. Data obtained were imported to R statistical language [90] for further analysis and representation using phyloseq [91] and ggplot2 [92] packages.

### 4.4. Antimicrobial Susceptibility Assay

Antimicrobial susceptibility testing of 14 isolates was performed using the disk diffusion method described by Hudzicki, 2009 [93]. Mueller–Hinton I agar (DIFCO) was employed to evaluate bacterial susceptibility to six antibiotic drugs: cefotaxime (30 µg), ampicillin/sulbactam (10/10 µg), sulfamethoxazole/trimethoprim (1.25/23.75 µg), ciprofloxacin (5 µg), imipenem (10 µg). Zone inhibition diameters were interpreted according to CLSI breakpoint tables [94]. All studies were carried out in triplicate. The halo measurements were expressed as the average of the measurements plus the standard deviation. *Escherichia coli* ATCC© 25922 was used as a quality control strain.

## 5. Conclusions

Our study provides valuable insights into the prevalence, diversity, and nature of antimicrobial resistance genes presented in environmental water recovered from a lake system enclosed beside a city. By elucidating the dynamics of ARG and its dissemination, we can contribute to the collective efforts aimed at combatting the occurrence of resistance phenomena and preserving the efficacy of antimicrobial agents for future generations. Research on the identification of antimicrobial resistance and virulence genes in environmental water highlights the urgent need for standardized monitoring methods to address the global public health threat posed by antibiotic resistance. Understanding the presence, diversity, and transmission pathways of resistance genes in water environments is essential for developing effective strategies to mitigate the spread of antimicrobial resistance between microbial species and the generation of antimicrobial multidrug-resistant microorganisms.

## Figures and Tables

**Table 1:** Summary of antibiotic resistance genes found and associated resistance mechanisms.

Family Gen	Antibiotic Family	Drug Example	Resistance Mechanism Example	Literature References
*bla*	Beta-lactams	Imipenem	Antibiotic Inactivation	[24]
*cat*	Phenicols	Chloramphenicol	Antibiotic Inactivation	[29]
*cfx*	Cefamycins	Cefoxitin	Antibiotic Inactivation	[31]
*dfr*	Diaminopyridines	Trimethoprim	Target modification	[26]
*erm*	Macrolides	Erythromycin	Target modification	[32]
*inu*	Lincosamides	Clindamicin	Antibiotic Inactivation	[33]
*mef*	Macrolides	Erythromycin	Efflux Pump	[34]
*msr*	Macrolides	Erythromycin	Efflux Pump	[35]
*oqx*	Multi-Drug	Multi-Drug	Efflux Pump	[36]
*tet*	Tetracyclines	Doxicycline	Efflux Pump, Target Modification, Antibiotic Inactivation	[23]
*vat*	Streptogramins	Virginiamycin	Antibiotic Inactivation	[37]

**Table 2:** Susceptibility studies on bacteria isolated from Llanquihue Lake.

nº	Species	Antibiotic Drug Tested											
		Cefotaxime		Ampicillin /Sulbactam		Sulfamethoxazole /Trimethoprim		Gentamicin		Ciprofloxacin		Imipenem	
		IZD (mm)	Int	IZD (mm)	Int	IZD (mm)	Int	IZD (mm)	Int	IZD (mm)	Int	IZD (mm)	Int
23	*Citrobacter freundii*	36.3 ± 0.6	S	19.7 ± 0.6	S	26.0 ± 1.0	S	19.3 ± 0.6	S	39.0 ± 1.0	S	28.3 ± 1.5	S
55	*Citrobacter gillenii*	36.3 ± 1.5	S	32.0 ± 1.0	S	24.3 ± 0.6	S	24.0 ± 1.0	S	44.3 ± 1.2	S	27.3 ± 0.6	S
62	*Citrobacter gillenii*	32.7 ± 1.2	S	38.0 ± 2.0	S	22.7 ± 0.6	S	20.7 ± 0.6	S	50.0 ± 1.0	S	37.0 ± 1.7	S
2	*Enterobacter absuriae*	34.7 ± 2.9	S	29.7 ± 0.6	S	33.3 ± 0.6	S	19.3 ± 1.5	S	36.3 ± 0.6	S	27.3 ± 0.6	S
14	*Enterobacter cloacae*	35.7 ± 1.2	S	30.0 ± 0.0	S	32.7 ± 1.2	S	19.7 ± 1.2	S	40.3 ± 0.6	S	31.0 ± 1.0	S
39	*Enterobacter ludwigii*	34.0 ± 1.7	S	32.3 ± 1.2	S	31.3 ± 0.6	S	23.3 ± 0.6	S	47.3 ± 0.6	S	33.0 ± 1.0	S
41	*Enterobacter ludwigii*	35.0 ± 2.0	S	34.7 ± 0.6	S	35.7 ± 0.6	S	24.0 ± 0.0	S	49.0 ± 1.0	S	35.3 ± 1.2	S
21	*Escherichia coli*	36.7 ± 0.6	S	19.7 ± 0.6	S	24.7 ± 0.6	S	24.3 ± 0.6	S	34.0 ± 0.0	S	31.0 ± 1.0	S
22	*Escherichia coli*	35.3 ± 06	S	20.3 ± 1.2	S	26.3 ± 0.6	S	20.3 ± 0.6	S	41.3 ± 1.2	S	31.3 ± 1.5	S
26	*Escherichia coli*	37.3 ± 0.6	S	23.3 ± 0.6	S	28.3 ± 2.1	S	22.0 ± 1.0	S	38.3 ± 0.0	S	29.3 ± 1.5	S
27	*Escherichia coli*	34.0 ± 1.0	S	20.0 ± 0.0	S	27.0 ± 1.0	S	19.7 ± 2.1	S	35.3 ± 0.6	S	31.3 ± 1.5	S
28	*Escherichia coli*	33.0 ± 0.0	S	21.3 ± 1.5	S	27.3 ± 0.6	S	23.7 ± 0.6	S	41.0 ± 1.0	S	32.3 ± 1.5	S
42	*Escherichia coli*	36.7 ± 1.5	S	20.3 ± 1.2	S	24.7 ± 0.6	S	19.0 ± 1.0	S	38.3 ± 0.6	S	30.3 ± 2.3	S
3	*Rahnella aquatilis*	25.3 ± 3.1	S	18.7 ± 1.2	S	21.3 ± 1.5	S	15.3 ± 0.6	S	25.3 ± 0.6	S	23.3 ± 0.6	S
ATCC 25922	*Escherichia coli*	31.3 ± 0.6	✓	20.7 ± 0.6	✓	24.7 ± 0.6	✓	24.3 ± 0.6	✓	45.0 ± 0.0	✓	34.7 ± 0.6	✓

IZD: Inhibition Zone Diameter expressed in millimeters mean ± standard deviation, Int: interpretation according to CLSI breakpoints [39], S: susceptible, ✓: quality control-approved according to values defined by CLSI [39].

## Data Availability

The raw data produced from DNA sequencing in this study were deposited in the ENA-EMBL database under the accession number PRJEB76156 (https://www.ebi.ac.uk/ena/browser/view/PRJEB76156, accessed on 18 July 2024). Metagenomic data obtained from bioinformatics analysis can be found in Supplementary Files.

## Supplementary Materials

The following supporting information can be downloaded at: https://www.mdpi.com/article/10.3390/antibiotics13070679/s1, Figure S1: Heatmap of pathogen species identified in Llanquihue Lake; Figure S2: Sample locations on Llanquihue Lake. File S1: Fasta MGEs sequences obtained from PlaSquid. Table S1: Taxonomic assignation obtained from Kraken2. Table S2: MGEs identified from PlasSquid sequences. Table S3: ARGs identified on MGEs.
