# Supplementary figures and images for: Antimicrobial Resistance Elements in Coastal Water of Llanquihue Lake, Chile
Source: Antibiotics (Basel). 2024 Jul 22;13(7):679. doi: 10.3390/antibiotics13070679 (PMC11273793; doi:10.3390/antibiotics13070679)

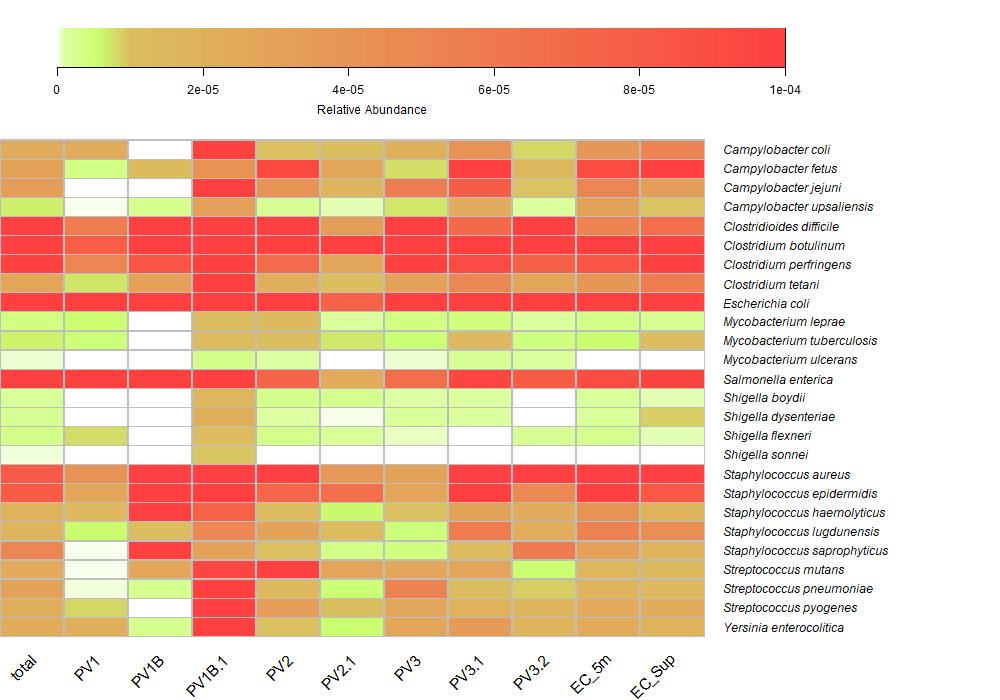

Supplement: Supplementary file 1 [file antibiotics-13-00679-s001.zip › Supplementary Figure S1.jpg]

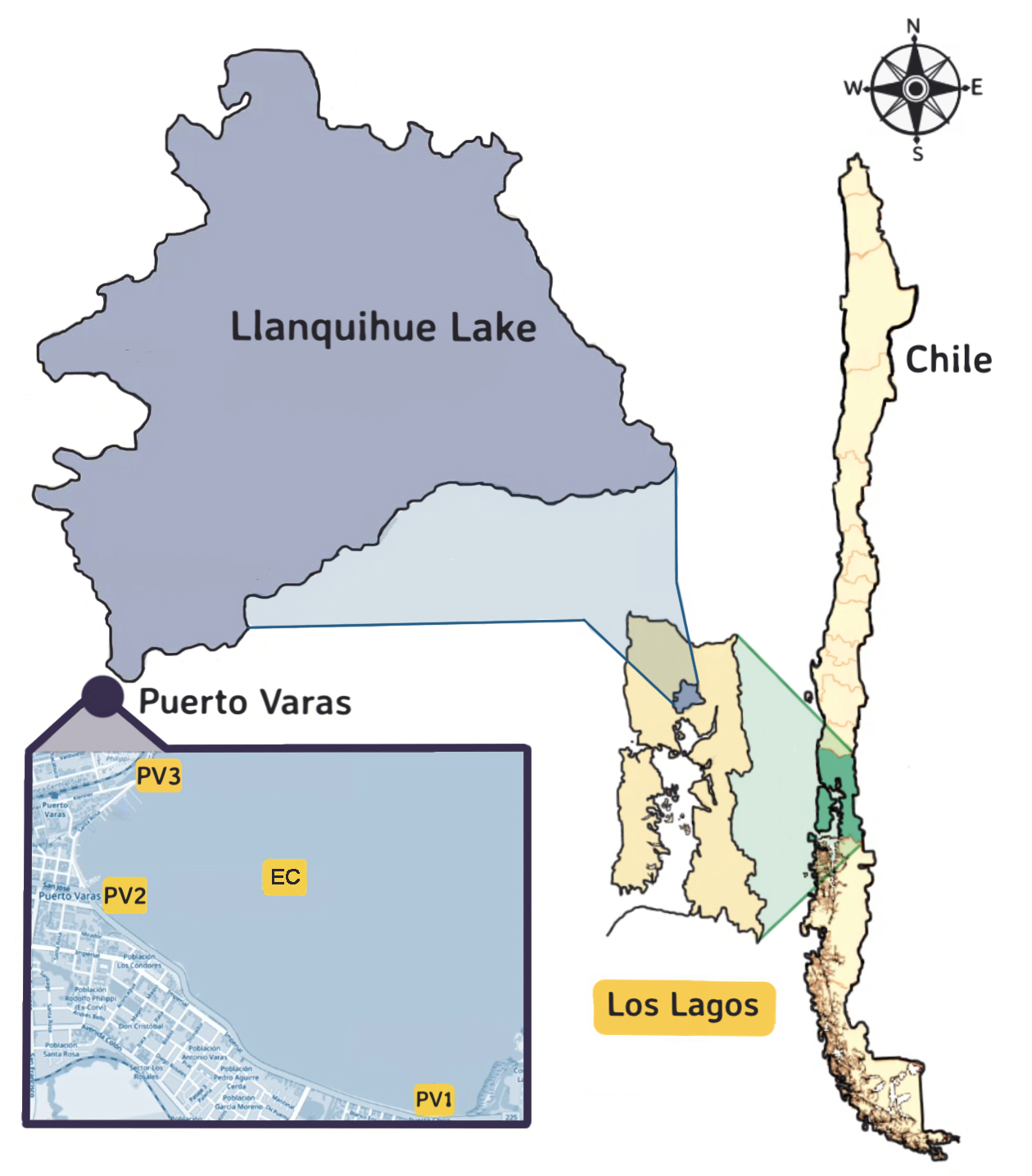

Supplement: Supplementary file 1 [file antibiotics-13-00679-s001.zip › Supplementary Figure S2.jpg]
